# Collecting initial accounts using ChatCharlie chatbot improves eyewitness memory in later investigative interviews

**DOI:** 10.1038/s41598-025-93281-1

**Published:** 2025-03-19

**Authors:** Coral J. Dando, Charlotte E. Adam

**Affiliations:** 1 Department of Psychology, University of Westminster, 115 New Cavendish Street, London, W1W 6UW UK; 2 Division of Psychology, University College London, London, UK

**Keywords:** Eyewitness memory, Initial accounts, Chatbots, Investigative interview, Delay, Memory consolidation, Human behaviour, Physical sciences

## Abstract

Initial account interviews (IAi) offer eyewitnesses more immediate opportunities to answer a series of brief questions about their experiences prior to an in-depth, more formal investigative interview. An IAi is typically elicited in-person near/at the scene of a crime using broadly systematic questioning. Retrieval practice can improve subsequent recall in some contexts, but there is a dearth of research centred on the potential costs and benefits of a quick IAi. Furthermore, where an in-person IAi is impossible, no alternative quick provision exists. Given the systematic nature of the IAi protocol, we developed a conversational chatbot as a potential alternative. Using a mock-witness paradigm, we investigated the memory performance of adults from the general population during in-depth in-person interviews one week after having provided an IAi 10 min post event either (1) in person, (2) via the ChatCharlie chatbot, or (3) no IAi (control). IAi conditions leveraged significantly improved event recall during later investigative interviews versus the Control. Accounts were more accurate and complete, and more correct information was remembered without increased errors indicating the potential of digital agents for IAi purposes Findings concur with predictions from theoretical understanding of episodic memory consolidation and the empirical eyewitness literature regarding the benefits of practice in some contexts.

## Introduction

Information provided by witnesses and victims of crime (henceforth witnesses) is fundamental for guiding the investigation of crime from the offset, and remains crucial during the prosecution process, and beyond^1,2^. However, witness memories are not objective records of events, but representative reconstructions of personal experiences which can be likened to physical trace evidence such as DNA and fingerprints in terms of fragility^3–5^. Accordingly, witness memory should be treated with care since episodic long-term memory is hard to access, mailable and error-prone^6,7^.

How ‘best’ to manage the elicitation of information from witnesses to maximise the quality and quantity of information recalled is an enduring challenge for front-line investigators^8–11^. In the UK and elsewhere, witnesses typically provide information about their experiences during an investigative interview conducted by a police officer or other investigative professional. While practices may vary according to jurisdiction, the term investigative interview typically refers to a formal in-depth interview that takes place at a police station, a witness’ home or other appropriate interview facilities^12^. Here, interviewers seek to elicit a detailed and accurate account using techniques that draw on cognitive and social psychological theory and empirical evidence to support witnesses to recall and verbalise their experiences^13,14^.

Investigative interviews necessarily occur sometime after an event so delays between encoding and retrieval are common, and have long been highlighted^13,15–17^. Delays are variable according to numerous factors including investigator availability, access to appropriate interview facilities, operational challenges, event and individual witness considerations and can range from a few hours, to months and even years later in some instances^12,15,17,18^.

The extant theoretical and applied memory literature is consistent in indicating the value of timely retrieval practice (where appropriate), since episodic information is less stable and so vulnerable to interference during the period after encoding. Encoding occurs rapidly, followed by the more gradual consolidation process of transforming fragile newly encoded episodic memories into long-term more stable forms^19–23^. Practice can support consolidation, helping to stabilize memory, making it more robust than might otherwise be the case, typically enhancing recall memory for practised items by supporting goal-directed episodic memory^20,21,23^. A timely initial retrieval also has potential to confer investigative benefits by leveraging important investigation relevant information at an earlier stage than might otherwise be the case.

In recognition of the potential benefits of a quick first retrieval, the College of Policing (CoP), the professional policing public body in England and Wales, has developed and implemented an initial account interview protocol. The Initial Account interview (IAi) is a ‘quick’ initial interview that takes place at or near the scene of the event in question, towards improving the accuracy and quantity of information provided by witnesses during a later investigative interview and supporting police to take timely and appropriate action quickly after an event (College of Policing, 2024).

In some instances, witnesses may be able to provide an initial statement about their experiences by completing a Self-Administered Interview (SAI). The SAI (available in paper-based and more recently digital formats)^15,24–26^ aims to elicit detailed accounts and so differs considerably to the IAi protocol in terms of rationale, implementation, complexity, retrieval techniques and questioning approach. The IAi elicits a very brief account of an incident to inform police of what happened and help them take immediate action at the scene. An IAi is short, context-dependent, and focuses on securing evidence, identifying offenders, safeguarding, assessing risk, and identifying offences. The SAI on the other hand gathers a more comprehensive initial statement, and so seeks to leverage detailed episodic retrieval, and thus is not countenanced for immediate action purposes. Although not the focus of the research reported here, nonetheless, the SAI literature offers significant evidence of the applied benefits of timely first retrieval.

Despite the extant theoretical and experimental research in support of the potential cognitive benefits of an IAi, as advocated by the CoP, there is a dearth of applied eyewitness memory research in this regard. The CoP approach for IAi has yet to be empirically evaluated with adults from the general population, and so the cognitive benefits are not well understood. Further, since fast initial accounts are provided in response to a series of questions posed by a police officer or other investigative professional, when this is not possible there appears little alternative but to forgo an IAi. In such instances the cost in terms of memory performance in later investigative interviews is unclear. A lack of appropriate alternatives in the face of finite human resources, or in contexts where gathering an initial account is inappropriate due to large numbers of witnesses for example, might prove costly for criminal justice processes.

The research reported here moves towards filling gaps in understanding by investigating the efficacy of a CoP style IAi on later recall during an in-depth investigative interview versus providing no initial account. Since the current CoP guidance presents a largely prescriptive approach to asking a series of straightforward initial questions, this type of interaction lends itself to some element of atomisation with potential for offering alternative provision^16,27–29^. Here we empirically evaluate a potential addition to the initial account ‘toolbox’ in the form of a remote contextual chatbot which we refer to as ChatCharlie. ChatCharlie has been designed to engage witnesses in a conversation style interaction in a manner that broadly maps onto the CoP initial account approach, and so we also consider the impact of a chatbot based IAi on recall one week later (see “Methods”).

ChatCharlie has potential to offer witnesses 24/7 access to a synchronous initial account digital agent to guide them in providing a timely initial account in line with current best practice for supporting episodic recall, and in a manner that is well aligned with the operational benefits of an initial account suggested by the CoP. The use of various types of chatbots (e.g., hybrid, AI, conversational) is rapidly expanding across numerous sectors, including education, financial services, and public health^30–32^, for example. The benefits of human–chatbot interactions include timely access to information and targeted assistance, improved self-disclosure in part resulting from the anonymous non-judgmental nature of human–chatbot interactions, and in some cases, chatbots have been found effective for providing social support.

Human–chatbot interactions are not without challenges. Poor user experiences are widely reported^33,34^, particularly when chatbots do not provide relevant answers to questions and so do not support users in resolving their queries or problems^35,36^, and when access is limited and interface design is poor^37,38^. User needs can be complex and so human–chatbot dialogue that is helpful and provides relevant and timely responses are key drivers to improving user experiences. For the purposes of the research reported here, in the absence of a human questioner, a suitably designed chatbot may offer a ‘next best’ option for triggering the process of extracting knowledge from personal experiences via IAi. One corollary being memory consolidation processes are supported and strengthened by ChatCharlie, resulting in quantifiable benefits and improved recall later versus the absence of an IAi practice when a human agent is unavailable.

ChatCharlie is not a customer support agent, but rather a conversational agent designed to collect and securely store textual responses to a series of contextually relevant questions. ChatCharlie has been designed to reduce perceptions of unresponsiveness by timing messages optimally^39–41^, ensuring witnesses are not overwhelmed by information and recall requests, but that response lag does not appear so unhuman like as to feel uncomfortable and/or frustrating. ChatCharlie offers users a human like name, which can increase perceptions of an authentic interaction^42^ especially when paired with an informal communication style). Reassurances regarding confidentiality, and if appropriate anonymity, can also increase engagement and acceptability and as such ChatCharlie immediately and then intermittently offers some information in this regard. Finally, interface feedback was considered towards maximising the likelihood of an acceptable user experience. Alpha testing of ChatCharlie included testing buttons, icons and input fields, considering the logical flow and understandability of the questions and information, and limit testing to establish ChatCharlie responses in situations of unforeseen response scenarios. ChatCharlie was refined based on Alpha testing responses and adjusted accordingly prior to this research, which represents Beta style testing.

The novelty of this research and a dearth of literature relevant to quick IAi style interview with adults and the use of chatbots in eyewitness memory contexts supported the formulation of the following hypotheses. As predicted by contemporary understanding of long-term memory, our first hypothesis H^1^ was that irrespective of method of initial account retrieval method (In-person; ChatCharlie), providing an IAi promptly would result in improved recall during a more in-depth In-person investigative interview one week later. Our second null hypothesis H^0^ was that there would be non-significant memory performance differences between ChatCharlie and In-person initial account retrievals, since ChatCharlie mimics the prevailing IAi protocol. Rapport building is countenanced by the CoP protocol and is present in the In-person initial account condition, but necessarily absent when initiating an initial account via ChatCharlie. Reductions in social demand inherent in interactions when humans are absent may counterbalance the absence of rapport in ChatCharlie. Further, attempts to build rapport would not be expected, and so likely viewed as ‘false’ and inappropriate when communicating with a digital agent, which can divert cognitive resources away from invoking episodic retrieval mode^43–45^.

## Results

To investigate H^1^ we conducted a series of multivariate and univariate analyses of Time 2 interview recall performance metrics (e.g., correct, errors, confabulations, accuracy and completeness), globally (that is, from start of the Time 2 investigative interview to the end) and as a function of interview phase (free recall; cued recall). We analysed self-reported confidence in the correctness of recall and errors at Time 1 as a function of IAi condition and then again at Time 2 across all conditions to understand self-reported confidence in memory performance. To test the null hypothesis (H^0^) we conducted Bayesian analyses across each of the Time 1 IAi performance metrics (correct, errors, confabulations) to understand the relative likelihood of a difference between ChatCharlie and In-person IAi memory performance at Time 1^46^. Additional exploratory analyses investigated self-report confidence in global correct and erroneous recall using t-tests and ANOVAs.

### Time 1 initial account

Bayesian tests (with default priors) revealed the amount of correct information recalled at Time 1 across the In-person and ChatCharlie IAi conditions did not significantly differ, *p* = 0.399, *d* = 0.22, BF _01_ = 3.71 (95% CI − 3.46, 8.39). Likewise, the number of errors and confabulation did not significantly differ, *p* = 0.237, *d* = 0.30, BF _01_ = 2.70 (95% CI − 0.56, 2.16) and *p* = 0.097, *d* = 0.43, BF _01_ = 1.44 (95% CI − 0.09, 0.96), respectively. See Table 1 for means, SDs and 95% CI).

**Table 1:** Means, SDs and 95% CI In-person and ChatCharlie Time 1 initial account memory performance (correct, errors and confabulations).

	Mean (SD) 95% CI		
	Correct	Error	Confabulation
In-Person	39.50 (13.08) 34.62, 44.38	4.73 (2.84) 3.67, 5.79	1.00 (1.23) 0.54, 1.46
ChatCharlie	37.03 (9.05) 33.65, 40.41	3.93 (2.32) 3.07, 4.80	0.68 (0.76) 0.31, 0.82

### Global recall performance at time 2

Multivariate Analysis of Variance for global composite correct, erroneous and confabulated recalled during Time 2 investigative interviews (from start to finish) was significant, *F*(6, 172) = 9.680, *p* < 0.001, η_p_^2^ 0.25 (Pillai’s Trace). Follow-up discriminant analysis revealed two significant discriminant functions. The first explained 75.4% of the variance, conical *R*^2^ = 0.35, the second explained 24.6%, conical *R*^2^ = 0.15. In combination, both discriminant functions significantly differentiated the Time 2 retrieval groups, Λ = 0.55, χ^2^ (6) = 51.623, *p* ≤ 0.001 and Λ = 0.85, χ^2^ (2) = 14.131, *p* ≤ 0.001. Correlations between outcomes and the discriminant functions revealed that correct recall loaded highly onto both functions (*r* = 0.64 and *r*.72, respectively). However, confabulations loaded more highly into the first function (*r* = 0.46) which explained 75.4% of the variance than in function 2 (*r* = − 0.60), whereas errors were loaded more highly into the second function (*r* = 0.15) than in the first function (*r* = − 0.25), which explained just 24.6% of the variance. Correct recall combined with confabulations significantly discriminated the Control from ChatCharlie and In-person Time 1 conditions. Correct recall combined with errors significantly discriminated ChatCharlie from both the Control and In-person Time 1 conditions.

Univariate *F*s for each of the Time 2 dependent variables (followed by post hoc Games-Howell tests (applying Bonferroni’s correction as appropriate) revealed a significant main effect of condition for correct recall at Time 2, *F*(2, 87) = 13.935, *p* < 0.001, η_p_^2^ 0.24. Participants in the ChatCharlie and In-person Time 1 conditions recalled more correct information at Time 2 than those in the Control condition, all *p*s < 0.001. There was a non-significant difference between the former two conditions, *p* = 0.256 (see Table 2).

**Table 2:** Time 2 mean global memory performance measures and percentage accuracy as a function of Time 1 initial account condition (*N* = 90).

	Means (SD) 95% CI			
	Correct	Errors	Confabulations	% Accuracy
ChatCharlie	56.67 (16.05) 51.50, 61.83	6.57 (4.01) 5.26, 7.71	0.87 (0.86) 0.47, 1.26	88.94 (4.61) 86.90, 90.97
In-Person	52.47 (14.40) 47.30, 57.63	4.40 (1.69) 3.26, 5,54	1.63 (1.16) 1.24, 2.03	89.87 (4.71) 87.84, 91.90
Control	38.17 (12.00) 33.00, 43.33	5.97 (3.28) 4.83, 7.11	0.73 (1.20) 0.34, 1.13	85.24 (7.11) 83.21, 87.27

#### Erroneous recall

There was a non-significant main effect of condition for the amount of erroneous recall at Time 2, *F*(2, 87) = 3.791, *p* = 0.026, η_p_^2^ 0.08 (see Table 2).

#### Confabulations

There was a significant main effect of condition for the number of confabulations at Time 2, *F*(2, 87) = 6.019, *p* = 0.004, η_p_^2^ 0.12. Participants in both the In-person and ChatCharlie Time 1 conditions reported fewer confabulations at Time 2 than those in the Control, *p* = 0.002 and *p* = 0.007, respectively. There was a non-significant difference between the former two conditions, *p* = 0.635 (see Table 2).

#### Percentage accuracy

Analysis of variance revealed a significant main effect for percentage accuracy in Time 2 interviews, *F*(2, 87) = 180.126, *p* < 0.001, η_p_^2^ 0.12 (see Table 1 for means SDs and 95% CIs). Participants in the Control condition were less accurate at Time 2 than those in both the In-person and ChatCharlie Time 1 conditions, *p* = 0.006 and *p* = 0.037, respectively, with a non-significant difference between the ChatCharlie and In-Person IAi conditions, *p* = 0.718 (see Table 2).

#### Completeness

The template approach used for coding Time 2 interviews comprised 111 items of event information. Completeness was the amount of correct recalled as a percentage of 111. There was a significant difference across conditions for completeness at Time 2, *F*(2, 87) = 13.925, *p* < 0.001, η_p_^2^ 0.24. Participants in the Control condition were significantly less complete (*M*_Control % complete_ = 34.38, SD = 18.81, 95% CI 30.35, 38.42) than those in the ChatCharlie and In-person conditions, all *p*s < 0.001 (*M*_ChatCharlie % complete_ = 51.05, SD = 14.42, 95% CI 45.67, 56.44; *M*_In-person % complete_ = 47.27, SD = 12.97, 95% CI 42.42, 52.11), with no significant difference between the latter two conditions, *p* = 0.537.

### Time 2 interview phase memory performance

#### Free recall

**Fig. 1:**
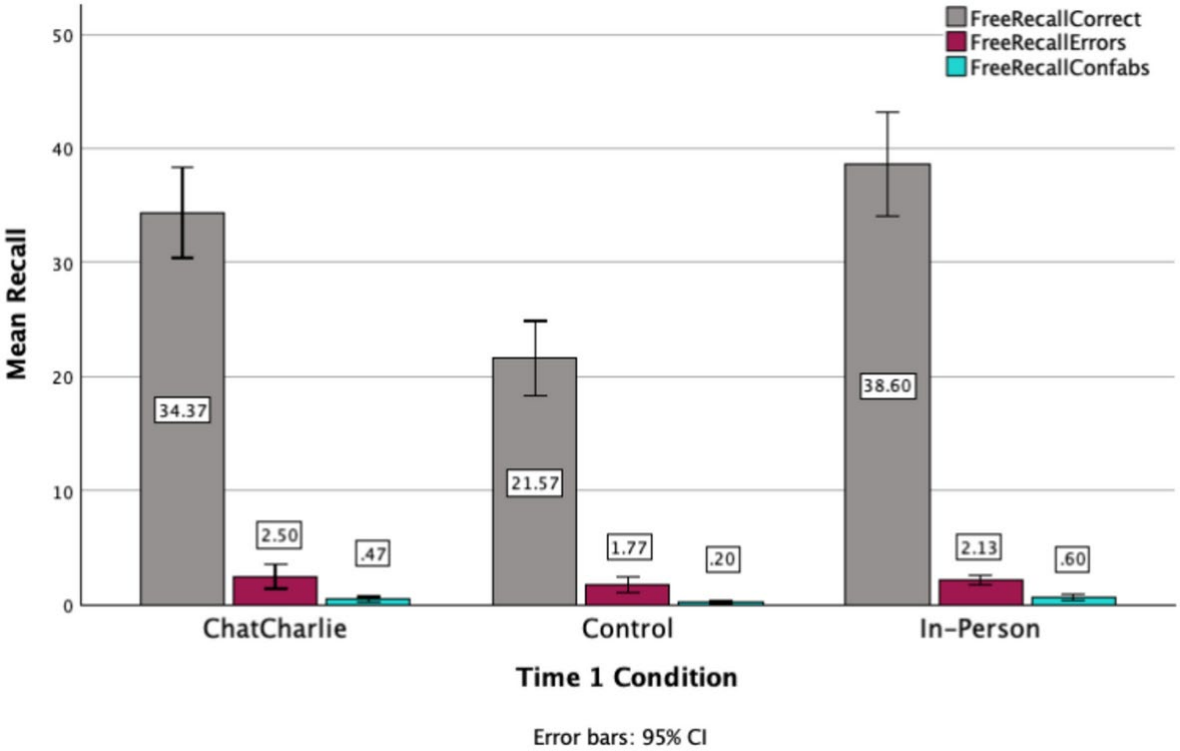
Mean recall for correct, errors and confabulations in the free recall phase of Time 2 interviews as a function of Time 1 retrieval condition (*N* = 90).

There was a significant main effect for correct recall in the free recall phase of Time 2 interviews, *F*(2, 87) = 20.786, *p* < 0.001, η_p_^2^ 0.32 (see Fig. 1). Participants in the Control recalled significantly fewer correct information items than those in both ChatCharlie and In-person Time 1 conditions, all *p*s < 0.001, with a non-significant difference between the latter two conditions, *p* = 0.718. There were non-significant differences for errors and confabulations, *F*(2, 87) = 0.932, *p* = 0.397, η_p_^2^ 0.02, and, *F*(2, 87) = 3.053, *p* = 0.052, η_p_^2^ 0.07, respectively.

There was a significant difference across conditions for percentage accuracy, *F*(2, 87) = 7.572, *p* < 0.001, η_p_^2^ 0.15. Participants in the ChatCharlie condition were more accurate (*M* = 92.66, SD = 5.74, 95% CI 89.99, 95.34) than participants in and Control (*M* = 92.09, SD = 7.15, 95% CI 89.41, 94.77), *p* = 0.003 and In-person (*M* = 85.97, SD = 8.90, 95% CI 83.30, 88.65) conditions, *p* = 0.013, with non-significant differences between the former two conditions, *p* = 0.937.

#### Cued recall

**Fig. 2:**
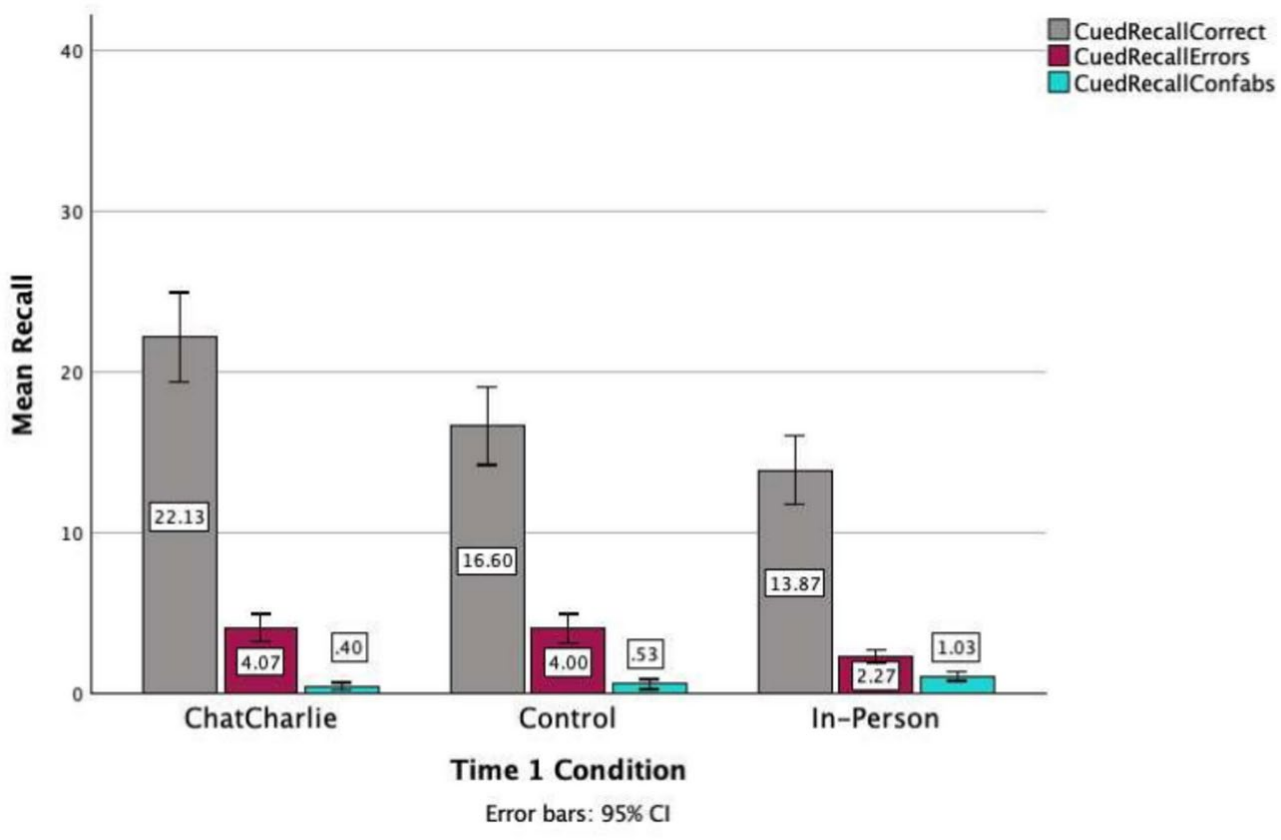
Mean recall for correct, errors and confabulations in the cued recall phase of Time 2 interviews as a function of Time 1 retrieval condition (*N* = 90).

There was a significant main effect for the number of correct information items recalled in the cued recall phase at Time 2, *F*(2, 87) = 12.215, *p* < 0.001, η_p_^2^ 0.22. Participants in ChatCharlie recalled significantly more correct information than participants in both the Control and In-person conditions, *p* = 0.009 and *p* < 0.001, respectively, with no difference between the latter two conditions, *p* = 0.204 (see Fig. 2). There was a significant difference for errors, *F*(2, 87) = 7.641, *p* < 0.001, η_p_^2^ 0.22. Participants in the In-person condition recalled significantly fewer errors than Control and ChatCharlie, *p* = 0.003 and *p* < 0.001, respectively, with a non-significant difference between the latter two conditions, *p* = 0.993.

There was a significant difference across conditions for confabulations in the cued recall phase of Time 2 interviews, *F*(2, 87) = 5.635, *p* = 0.005, η_p_^2^ 0.11. Participants in the In-person Time 1 condition confabulated more during the cued recall phase at Time 2 than participants in ChatCharlie condition, *p* = 0.004. All other differences were non-significant, all *ps* > 0.061 (see Fig. 2).

There was a significant difference in percentage accuracy in the cued recall as a function of Time 1 condition, *F*(2, 87) = 6.581, *p* = 0.002, η_p_^2^ 0.13. Participants in ChatCharlie (*M* = 84.12, SD = 6.96, 95% CI 79.08, 89.15) were significantly more accurate than those in the In-person Time 1 condition (*M* = 71.14, SD = 19.79, 95% CI 66.12, 76.18), *p* = 0.005. There was a non-significant difference between the control (*M* = 78.27, SD = 11.70, 95% CI 73.24, 83.30) and ChatCharlie, *p* = 0.59.

### Memory confidence

Self-reported confidence was gathered from participants in both IAi conditions immediately post completion of an IAi (at Time 1) and from all participants immediately after the Time 2 interviews. It is not uncommon to collect self-report global confidence in this manner so as not to interfere with, and potentially truncate, the retrieval process (e.g., Odinot & Wolters, 2006; Dando et al., 2023). Interrupting retrieval to variously collect confidence data following responses to each question in turn may be of import for theoretical and confidence-accuracy calibration purposes, but here we were more concerned with mimicking the real world and thus employing witness-focused retrieval techniques when collecting confidence measures.

#### Confidence Time 1

There was a significant difference between ChatCharlie and In-person initial accounts conditions for self-reported confidence (ranging from 1 = not at all confident to 5 = completely confident) in the amount of correct event information recalled at Time 1, *t* (58) = 8.056, *p* < 0.001,* d* = 1.04. Participants in the In-person condition were more confident that they had remembered a lot of correct information (*M*_In-person_ = 3.47, SD = 0.860) than ChatCharlie participants (*M*_ChatCharlie_ = 2.80, SD = 1.19). There was a non-significant difference between ChatCharlie and In-person conditions for self-reported confidence in the number of errors recalled at Time 1, *t* (58) = 4.409., *p* = 0.040 (*M*_In-person_ = 3.67, SD = 0.880; *M*_ChatCharlie_ = 2.80, SD = 1.37).

#### Confidence Time 2

There was a significant main effect for self-reported confidence in the amount of correct event information recalled at Time 2, *F*(2, 87) = 18.286, *p* < 0.001, η_p_^2^ 0.29. Participants in the In-person IAi condition were significantly less confident at Time 2 than those in both the ChatCharlie and Control conditions, all *p*s < 0.001, with a non-significant difference between the latter two conditions, *p* = 0.945.

There was also a significant main effect for how confident participants were that they had not made any errors at Time 2, *F*(2, 87) = 11.694, *p* < 0.001, η_p_^2^ 0.21. Participants in the In-person IAi condition were more confident that they had not made any errors at Time 2 than those in both the ChatCharlie and Control conditions, all *p* = 0.003 and *p* < 0.001, respectively. There was a non-significant difference between the latter two conditions, p = 0.458.

#### Confidence Time 1 to Time 2

**Fig. 3:**
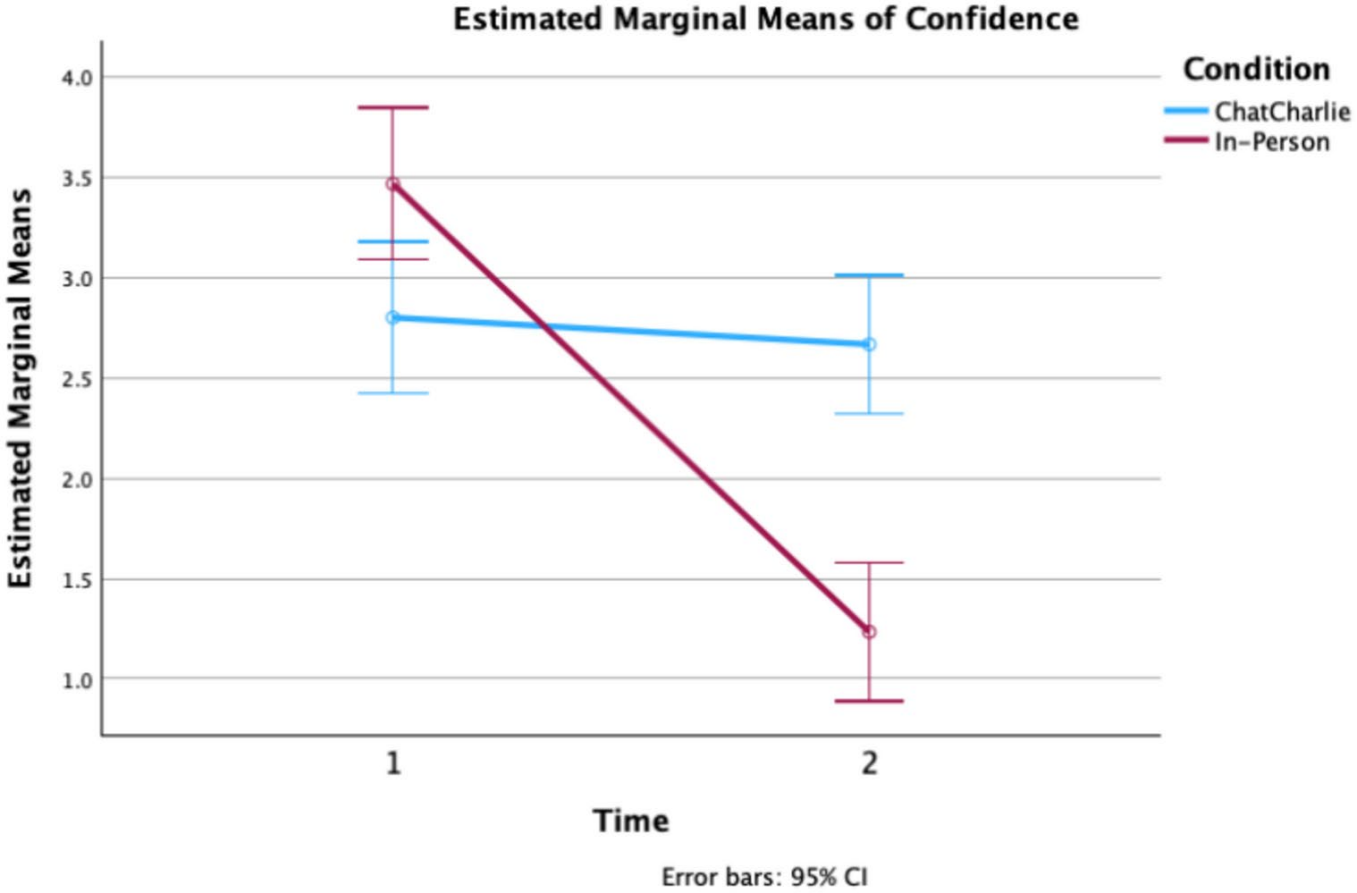
Confidence in correct recall across retrieval attempts (from Time 1 to Time 2) as a function of ChatCharlie and In-Person Time 1 conditions (*N* = 60).

Repeated measures analysis revealed a main effect of Time for self-reported confidence in correct information, *F* (1, 58) = 66.906, *p* ≤ 0.001, η_p_^2^ 0.54, and a significant Time X Condition mixed interaction, *F* (1, 58) = 52.678, *p* ≤ 0.001, η_p_^2^ 0.48. Irrespective of Time 1 condition (ChatCharlie; In-person), confidence in the amount of correct information recalled dropped from Time 1 (*M* = 3.13, SD = 1.08, 95% CI, 2.866, 3.033) to Time 2 (*M* = 1.95, SD = 0.86, 95% CI 2.051, 2.649) at Time 2. Confidence of participants in the In-person Time 1 condition dropped significantly from Time 1 to Time 2, *p* < 0.001, whereas confidence of participants in ChatCharlie remained constant from Time 1 to Time 2, *p* = 0.517 (see Fig. 3).

There was a non-significant repeated measure main effect for confidence in errors from Time 1 (*M* = 3.08, SD = 1.17) to Time 2 (*M* = 2.92, SD = 1.02) and a non-significant Time X Condition mixed interaction, *Fs* < 1.495, all *p*s > 0.226 (*M*_ChatCharlie T1_ = 2.50, SD = 1.14; *M*_ChatCharlie T2_ = 2.55, SD = 1.00; *M*_In-person T1_ = 3.67, SD = 0.88; *M*_In-person T 2_ = 3.33, SD = 0.84).

#### Confidence and accuracy

Regression analysis indicated that Time 1 and Time 2 self-reported confidence for correct and erroneous information were non-significant predictors of accuracy at Time 2, *F*(2, 59) = 0.741, *p* = 0.481, and, *F*(2, 59) = 1.133, *p* = 0.329, respectively accounting for only 2.5% and 7.6% in the variation of percentage accuracy at Time 2, respectively.

## Discussion

IAi interviews offer witnesses an immediate opportunity to briefly explain their experiences. They are not appropriate in all instances nor for all witnesses, and IAis do not replace in-depth investigative interviews nor SAIs but rather precede both if appropriate. Nonetheless, IAIs offer the opportunity for witnesses to practice in a managed fashion, potentially stabilizing eyewitness memory, making it more robust than might otherwise be the case. Irrespective of Time 1 IAi retrieval mode (In-person or ChatCharlie), global memory performance during investigative interviews at Time 2 (one week later) was significantly improved.

Our results offer clear support for H^1^, thus providing additional evidence of the benefits for eyewitnesses of providing an initial account promptly^15,24^. Participants who ‘practised’ via an IAi at Time 1 recalled 37% (In-person) and 47% (ChatCharlie) more correct information at Time 2, versus those who did not provide an IAi. Confabulations were reduced and significant improvements in episodic recall were not accompanied by an increase in errors, thus accuracy was higher indicating positive verbal and textual initial retrieval practice effects. Others have reported similar benefits following a SAI^15,24,47^ which was designed to support comprehensive retrieval attempts akin to an in-person investigative interview (if appropriate) but without the need for an interviewer. The IAi is not a replacement for an SAI, nor vice versa, since they have been designed and developed for different purposes and the SAI does not capture a ‘quick’ account in line with current CoP guidance. Nonetheless, in instances where the SAI has also been used, subsequent recall has been found to be more accurate and detailed.

ChatCharlie is a conversational question–answer digital agent to guide witnesses in providing a quick initial account (if appropriate) when human agents are unavailable. ChatCharlie is akin to the verbal in-person IAi currently advocated in terms of investigative approach, although rapport building is necessarily absent^48,49^. Yet, ChatCharlie appears equally as effective since Time 2 global correct recall performance was improved versus the Time 1 control condition (no IAi) with the Time 1 centred null hypothesis being more likely than the alternative hypothesis.

The pattern of results as a function of the individual retrieval phases typically present in basic investigative interviewing practices in the UK and elsewhere is more nuanced and worthy of consideration. Global interview findings are mirrored in the initial free recall phase, but performance in the second cued questioning retrieval reveals ChatCharlie participants reported almost 60% more correct information at Time 2 and confabulated significantly less than In-person IAi participants. However, ChatCharlie participants verbalised more errors (partially correct information) in this phase. The number of errors were few and the mean difference is small, and because ChatCharlie participants reported fewer confabulations they are more accurate in this phase. Nonetheless, this difference in errors was significant.

One potential, albeit partial explanation for the pattern of results in the second recall phase of Time 2 interviews emerges from in-person interviewing contexts and may have been triggered by the move from person absent interview contexts to In-person interviewing. Real and/or perceived demand^50^ has the potential to inflate confidence and can encourage responses to questions resulting in the reporting of erroneous information in face-to-face interviews^51–54^. The absence of a human interviewer can support more cautious remembering, thus reducing errors and so our findings may be social in nature, rather than cognitive^16,55^. However, more research focusing on the potential impact of moving from an initial account interview with a digital agent to an in-person investigative interview is necessary to fully understand the locus of this effect.

Our null hypothesis was also supported with non-significant IAi memory performance differences at Time 1 across the ChatCharlie and In-person conditions. We considered the potential costs and benefits of no rapport when engaging with a digital agent^16,45,55^. Demand characteristics can be amplified in ‘performance’ contexts as is the case in applied witness memory research and so here, Time 1 performance across both Time 1 conditions suggests the trade-off between the presence/absence of a human agent and presence/absence of rapport appears acceptable in terms of memory performance. Further research regarding the investigative relevance of recalled information at Time 1 would improve understanding, as would more nuanced manipulation of rapport across conditions. However, given the prescriptive ‘getting down to business’ nature of an initial account interview when a human agent is unavailable our findings indicate ChatCharlie may be appropriate in some instances.

Self-report confidence did not predict memory accuracy, so despite experimental control in mock witness laboratory research resulting in ‘pristine’ conditions, predictive confidence judgments shortly after the event at Time 1 and again at Time 2 were not ‘useful’ performance indicators. We concur with others in that, at best, in applied contexts recall confidence should be treated with caution, particularly following in-person interviews where confidence may be inflated for reasons previously discussed centred on social cognition and perceived performance demand. A well-managed in-person interview is by its very nature witness-focused and aims to support witnesses to feel socially comfortable. Feeling socially comfortable may boost confidence in one’s performance^56,57^. Although speculative and so further research is necessary, here the In-person initial account protocol included rapport building and did leverage significantly higher levels of confidence at Time 1, which dropped significantly from Time 1 to Time 2. ChatCharlie confidence levels were lower from the offset but remained stable across time points.

Best practice and investigative training emphasise the need for caution centred on appropriate acceptance of responses to questions and considered verbal encouragement of effortful retrieval. However, witnesses can misinterpret appropriate encouragement, and over encouragement can occur, albeit this may be subtle in nature or unintended since interviewing is cognitively demanding for interviewers and so behavioural leakage can occur. Confidence has been found to decline in conditions of real stimuli and repeat confidence checking^58^. Although we did not repeat check confidence within interviews, for participants in the In-person and ChatCharlie Time 1 conditions, we did collect confidence data at Time 1 and again at Time 2. However, it is unclear why a repeat check might trigger a drop in confidence across the two time points for In-person Time 1 participants but not for ChatCharlie Time 1 participants, and why in the latter condition confidence remained constant. Again, there is significant scope for further applied confidence, accuracy and digital versus human agent research.

Our research is novel in terms of investigating the current guidance on gaining an initial account in England and Wales. However, findings are limited on serval counts. Our sample size was sufficient to achieve 80% power for a medium effect for H^1^ and is in line with sample size norms described in recent similar research but may lack power to robustly reject the null hypothesis. Further, we have reported the benefits of retrieval practice, but practice can sometimes negatively interfere with later recall, particularly recognition performance. For example, providing a verbal description of a perpetrator prior to identifying the perpetrator from a line-up can compromise recognition accuracy in some instances. Referred to as verbal overshadowing^59^, this retrieval-based interference appears robust for forensic recognition tasks, where describing a visual experience can interfere with subsequent visual recognition^60,61^, although not always^62,63^.However, the IAi paradigm employed here was followed by an in-depth investigative interview retrieval that did not include a recognition task. Nonetheless, future research may wish to investigate IAi in terms of verbal overshadowing.

Retrieval-induced forgetting (RIF) offers an example of when retrieval practice can trigger the forgetting of related information at a later stage that was not previously recalled from the same information category^64^. The applied eyewitness RIF literature is very mixed whereby repeated questioning has been found to strengthen recall of certain event details while inducing the forgetting of other details in some contexts, but not always and is often context dependent^65,66^. Furthermore, different event details seem variously vulnerable to RIF in some instances^67,68^, and retrieval effort in the form of multiple recall attempts can predict memory accuracy over time sometimes resulting in improved recall with higher confidence in correct recall performance^69^. We did not investigate the possibility that an IAi might trigger RIF in adults, hence future research may also wish to consider the impact of an IAi on this phenomenon.

This research presents one study from a wider ongoing research programme concerning IAi and computer-mediated communication in investigative contexts more generally, and so the sample size and novelty limits generalisation. ChatCharlie is a prototype which will evolve, and maybe an AI version of ChatCharlie could incorporate some form of rapport which was absent here. Acceptability of ChatCharlie was not considered, yet acceptability for organisations and individuals is fundamental when considering the use of Chatbots in criminal justice contexts.

ChatCharlie may not emerge as the most desired method of providing an initial account for some groups, but in the absence of a human agent may be acceptable to many. Other witness populations may indicate a preference for this form of interaction since there is real potential for our ChatCharlie prototype to be more user-centred in terms of adjustment for neurodivergent eyewitnesses, for example. We did not consider the type of information recalled. Quantity and quality are important metrics in the first instance, but type of information and investigation relevance should be considered in the future, which would be relevant for researchers concerned with verbal overshadowing and RIF. Finally, mock witness research cannot replicate the real-world conditions experienced by witnesses but does offer an important opportunity to understand episodic memory in controlled conditions providing an indication of eyewitness cognition.

To conclude, a timely retrieval practice by way of responding verbally to initial account questions asked by a person or responding textually via a carefully managed contextual chatbot platform apparently offers benefits since in both instances later recall during an in-depth investigative interview was more detailed and more accurate. Our findings concur with the positive outcomes reported by others where witnesses are offered well-managed practice opportunities, providing empirical support for the cognitive benefits of eliciting initial accounts. We are not suggesting ChatCharlie is a replacement for professional skilled interviewers, rather we are exploring the potential of contextual conversational chatbots as an investigative and cognitive support tool in some instances.

## Methods

### Design

A between-subjects mock eyewitness paradigm with two distinct time points, namely Time 1 (IAi according to condition) and Time 2 (investigative interview) was employed. At Time 1 participants were randomly allocated to one of three Time 1 IAi conditions: ChatCharlie (conducted remotely via the ChatCharlie platform), In-person (conducted in-person) or Control (no initial account). At Time 2 (1 week later), all participants were then interviewed in-person, face-to-face using the same information gathering investigative interview protocol. Memory performance for the ChatCharlie and In-person IAi conditions were analysed at Time 1. Memory performance of all participants across conditions was analysed at Time 2. Self-reported confidence in correct and erroneous recall performance was collected at Time 1 (as appropriate) and Time 2.

### Participants

Ninety participants from the general population took part in this study: 28 male (31%), 60 female (67%), and 2 non-binary/third gender (2%). The mean age was 29.26 (SD = 8.01), ranging from 18 to 54 years. There was a non-significant difference in age across each of the three conditions, *F*(2, 87) = 2.128, *p* = 0.125 (*M*_ChatCharlie_ = 30.83; *M*_Control_ = 30.10; *M*_In-person_ = 26.83). Participants were recruited through online advertising and snowball sampling and were compensated for the time spent taking part through a £10 shopping voucher.

An *a-priori* power analysis was conducted using G*Power 4^70^ to determine the minimum sample size estimation to test H^1^. Power analysis for MANOVA: special effects and interactions indicated the required sample size to achieve 80% power for a medium effect at a significance criterion of *a* = 0.05 was *N* = 87. Thus, the obtained sample size of *N* = 90 was adequate given resource constraints and access to non-student populations and is in line with sample size norms described in recent similar empirical mock eyewitness studies^71,72^.

### Ethics

This research was ethically approved by the University of Westminster Research Ethics Committee, approval No ETH2021-0680. Experiments reported here were run in accordance with the British Psychological Society and UK Health Care Practitioner Council codes of ethical conduct, and the declaration of Helsinki. Written and digitally recorded verbal consent was obtained from each participant.

### Procedure

Participants consented to take part in both elements. Irrespective of Time 1 condition, all participants received a one-time link to watch a volume crime shop robbery on a laptop or desktop computer. Within 10 min of having viewed the event participants in ChatCharlie and In-person Time 1 conditions provided an initial account according to condition (see Appendices A & B) and completed an anonymous online questionnaire (see ESM Appendix C) regarding confidence in their memory performance (errors and correct recall). In-person initial accounts were elicited in-person face-to-face, and ChatCharlie initial accounts were elicited remotely via the ChatCharlie chatbot platform. Participants in the Control did not complete an initial account, nor a questionnaire at this timepoint. At Time 2 (one week later), all participants were interviewed In-person using a protocol, verbatim (ESM Appendix D). Immediately post Time 2 interviews, all participants completed an anonymous survey concerning confidence in their memory performance (ESM Appendix E). Participants were naive to the study design, aims and hypotheses.

### Materials

The stimulus video (accessible https://youtu.be/9MpWfQonSgk) was 1-min in duration and depicted a non-violent volume crime shop robbery involving two perpetrators who were seen walking past a parade of shops. They then entered a small corner store and after a short delay came running out of the shop chased by the shopkeeper. The perpetrators ran away from the shop and around the corner out of sight.

#### Initial account interviews (Time 1)

Both the In-person (see ESM Appendix A) and ChatCharlie (see ESM Appendix B) initial account Time 1 condition protocols draw on the CoP initial account guidance. Both focus on specific event details, individual and environmental factors and comprised ground rule instructions, open-ended invitations and closed and forced choice questions as appropriate. Confidence in correct recall was collected using a self report scale ranging from 1(not at all confident that the information I have reported is correct) to 5 (completely confident that the information I have reported is correct) at Time 1 and again at Time 2.   

#### Investigative interview (Time 2)

Time 2 investigative interviews were developed guided by the current approach to interviewing witnesses in the UK and elsewhere to best manage variability without limiting witness responses. Interviews comprised a series of distinct but seamless phases (see ESM Appendix C) namely (1) engage and explain, (2) retrieval instructions, (3) free recall, (4) cued questioning, and (5) closure. Of these, two were retrieval focused, the free recall and cued questioning phases. All Time 2 interviews were conducted by the same interviewer In-person.

### Recall performance coding

Time 1 In-person initial account interviews and all Time 2 investigative interviews were digitally audio and video recorded and transcribed verbatim. ChatCharlie conversation transcripts were downloaded from the chatbot platform. All transcripts were coded for correct, erroneous (information relevant to the witnessed episode but described with error, e.g., describing a person’s brown jacket, but stating that it was black instead of brown), or confabulated (reporting information that was not present in the film) information recalled. All information from the Time 1 initial account interviews and all information provided in the two retrieval phases of the Time 2 interviews, along with the position in the Time 2 interview the information was recalled was coded (i.e., whether recalled in the Free Recall or Questioning phase). Items recalled were only scored once (i.e., repetitions were not scored).

Fifteen Time 1 interviews and 15 Time 2 interviews from across the experimental conditions were randomly selected for recoding by an independent coder blind to the aims and hypotheses of the research but familiar with the method of scoring (RD). A series of Two-way mixed effects Intraclass Correlation Coefficient (ICC) analysis testing for absolute agreement between coders for the overall amount of correct, erroneous, and confabulated recall coding were conducted. Mean estimations with 95% CI reveal very good inter-rater reliability for Time 1 correct, ICC = 0.998 (95% CI 0.993, 0.999), errors, ICC = 0.922 (95% CI 0.766, 0.974) and confabulations, 0.944 (95% CI 0.835, 0.981) and Time 2 correct, ICC = 0.916 (95% CI 0.749, 0.972), errors, ICC = 0.840 (95% CI 0.524, 0.926) and confabulations, 0.921 (95% CI 0.765, 0.973). 

### Interviewer adherence to protocols

#### Time 1 in-person initial account interviews

Three interviewers (MZ; SA; CD) conducted the 30 In-person initial account interviews according to a protocol, verbatim (see ESM Appendix A). Interviewers conducted 11 (MZ), 10 (SA) and 9 (CD) first account interviews each. The first account protocol comprised a short introduction and rapport building phase followed by a series of fixed questions. A total of 6 (20%) initial account interviews (2 from each interviewer) were randomly selected and coded by two independent coders for interviewer adherence to the protocol for i) correct number of questions asked, ii) questions asked in the correct order, and iii) introductions and short rapport using a scoring sheet where 1 = absent, 2 = partially present and 3 = present, for each of the 3 protocol elements. Prior to coding, coders participated in a training session held by the first author, during which the initial account interview protocols and coding system were explained. Coders then practiced coding and discussed any disagreements/misunderstandings with the trainer to reach a consensus using example interviews. Two-way mixed effects intraclass correlation coefficient (ICC) analysis testing for absolute agreement between coders indicated good/very good inter-rater reliability for all interviewer behaviours: Kruskal–Wallis H tests for the presence/absence of each of the three interview instructions/phases revealed non-significant differences across the three interviewers, all *H*s (2) < 2.090, all *p*s > 0.598, hence all were similarly present/correct across interviewers.

#### Time 2

Time 2 interviews were all conducted by the same interviewer (CA). Eighteen interviews (20%) were randomly selected for interviewer adherence to the Time 2 protocol and coded by the same two independent coders from Time 1 using the same coding approach as Time 1 for each of the Time 2 protocol elements. Two-way mixed effects intraclass correlation coefficient (ICC) analysis testing for absolute agreement between coders indicated good/very good inter-rater reliability for the presence and application of each of the Time 2 interview phases: engage and explain, ICC = 0.938 (95% CI 0.843; 0.975), retrieval instructions, 1.00 (95% CI 1.00; 1.00), free recall, ICC = 0.920 (95% CI − 0.449; 0.362), questioning, ICC = 0.899 (95% CI 0.593; 0.975), and closure, ICC = 1.00 (95% CI 1.00; 1.00).

## Supplementary Information

Supplementary Information.

## Data Availability

Raw datafiles and materials are available from the first author and have been made available via the OSF: https://osf.io/ platform.
