# Supplementary material for: Collecting initial accounts using ChatCharlie chatbot improves eyewitness memory in later investigative interviews
Source: Sci Rep. 2025 Mar 19;15:9456. doi: 10.1038/s41598-025-93281-1 (PMC11923045; doi:10.1038/s41598-025-93281-1)
Supplement: Supplementary file 1 — Supplementary Information. [file 41598_2025_93281_MOESM1_ESM.docx]

**Appendix A: ChatCharlie IA Conversation Flow Protocol**

**Appendix B In-Person IA Protocol**

Hello, my name is XXXX. Thank you so much for agreeing to take part in this research we really appreciate it.

Quick rapport < 1 min

Now I am going to ask you some questions about the video you just watched. Do you have any questions before we start.

OK, if you are ready we will begin

To start, I want you to tell me everything you can remember about the video, in as much detail as possible. Whatever you can remember, even if you can only remember partial information. I want to know everything, but it is important that you do not guess. Only tell me what you remember.

Is there anything else you would like to add? Anything at all, even if it seems silly or unimportant.

How confident are you that this information is correct?

Was there anything obstructing your view?

Approximately how far away were you from what you saw?

When did this happen?

How many people did you see in the video?

I am now going to ask you some questions about the people/person, one at a time. In your mind label them person … (*person X, person X, person X*). *(depending on answer to previous question)*

Please describe person (number) in as much detail as possible.

Have you seen this person before?

Would you be able to recognise this person?

Can you describe their face?

What was this person wearing?

What was this person wearing on their top half?

What was this person wearing on their bottom half?

What was this person wearing on their feet?

Did they have anything with them?

Anything further you can tell me about this person? Anything at all, even if it seems silly or unimportant. I want to know everything.

How confident are you that this information is correct?

Repeat for each person, as appropriate.

Thank you. I think I now have a good idea of what has happened. Just before we finish is there anything else you want to tell me about what you saw, anything at all?

Is there anything you want to change about what you have already told me, anything at all?

Thank you that is the end of the interview.

**Appendix C**

Time 2 Investigative interview Protocol.

| **Phase** | **Overview** |
| --- | --- |
| 1.Explain | Explain the interview/research process prior to the commencement of the interview and offer the opportunity to ask questions. |
| 2.Rapport | Interviewer verbally interacts with the participant using two behaviours:   1. Open-ended invitations to exchange information. For example, 2. Offering some non-personal information about themself to begin this process*;*   Interviewer displays two attentive physical behaviours:   1. Looking at interviewees/making eye contact when they were talking. 2. Nodding when interviewees speak/answer questions.   Interviewer displays two attentive verbal behaviours:   1. Referring to the interviewee by their first name once the interviewee had agreed this would be acceptable.   ii) Thanking interviewees whenever they provided information and  answered a question. For example, *‘Thank you, that was useful*  *in helping me to understand’*  See Nahouli et al., 2021 and Dando et al., 2023 |
| 4.Free Recall | Commenced with an explanation of the four ground rules:   1. Report all/everything 2. Do not guess 3. Say if you do not know 4. Say if you do not understand   Participants were then instructed to explain everything they could remember, uninterrupted by the interviewer. The interviewer made bullet point notes regarding the topics recalled and the order in which they were recalled for use during the questioning phase. Once interviewees had finished, all were asked if they wished to add anything else. |
| 5.Cued Questions | Commenced with a reminder of the four ground rules (above), following which participants were asked one TED prefaced probing question cued by the topics recalled in the free recall, one by one. For example, *‘you mentioned a girl standing at the bar, please* ***describe*** *that girl to me in as much detail as you can’* |
| 6.Close | Participants were thanked and offered the opportunity to ask questions and make any alterations. |
